# Comparing Self-Reported Dietary Intake to Provided Diet during a Randomized Controlled Feeding Intervention: A Pilot Study

**DOI:** 10.3390/dietetics2040024

**Published:** 2023-11-17

**Authors:** James L. Casey, Jennifer L. Meijer, Heidi B. IglayReger, Sarah C. Ball, Theresa L. Han-Markey, Thomas M. Braun, Charles F. Burant, Karen E. Peterson

**Affiliations:** 1 Department of Nutritional Sciences, University of Michigan, Ann Arbor, MI 48109, USA; 2 Department of Molecular, Cellular, and Developmental Biology, University of Michigan, Ann Arbor, MI 48109, USA; 3 Department of Medicine, Dartmouth-Hitchcock Medical Center, Lebanon, NH 03756, USA; 4 Geisel School of Medicine, Dartmouth College, Hanover, NH 03755, USA; 5 Division of Metabolism, Endocrinology, and Diabetes, Department of Internal Medicine, University of Michigan, Ann Arbor, MI 48109, USA; 6 Department of Biostatistics, University of Michigan, Ann Arbor, MI 48109, USA

**Keywords:** 24 h dietary recall, dietary assessment, calories, protein, feeding intervention, systematic errors, random errors

## Abstract

Systematic and random errors based on self-reported diet may bias estimates of dietary intake. The objective of this pilot study was to describe errors in self-reported dietary intake by comparing 24 h dietary recalls to provided menu items in a controlled feeding study. This feeding study was a parallel randomized block design consisting of a standard diet (STD; 15% protein, 50% carbohydrate, 35% fat) followed by either a high-fat (HF; 15% protein, 25% carbohydrate, 60% fat) or a high-carbohydrate (HC; 15% protein, 75% carbohydrate, 10% fat) diet. During the intervention, participants reported dietary intake in 24 h recalls. Participants included 12 males (seven HC, five HF) and 12 females (six HC, six HF). The Nutrition Data System for Research was utilized to quantify energy, macronutrients, and serving size of food groups. Statistical analyses assessed differences in 24 h dietary recalls vs. provided menu items, considering intervention type (STD vs. HF vs. HC) (Student’s *t*-test). Caloric intake was consistent between self-reported intake and provided meals. Participants in the HF diet underreported energy-adjusted dietary fat and participants in the HC diet underreported energy-adjusted dietary carbohydrates. Energy-adjusted protein intake was overreported in each dietary intervention, specifically overreporting beef and poultry. Classifying misreported dietary components can lead to strategies to mitigate self-report errors for accurate dietary assessment.

## 1. Introduction

Estimating dietary intake is challenging due to random and systematic bias in self-reported diets. To quantify food intake, most researchers rely on memory-based dietary assessments, such as food frequency questionnaires (FFQ), food records, and 24 h dietary recalls, which have been debated over their value and validity [1,2]. Random and systematic errors that may occur during memory-based dietary assessments include (a) respondent systematically overreporting or underreporting foods [3]; (b) respondent unintentionally including or omitting foods [4,5]; and (c) respondent unable to recall portion sizes [6]. Several previous studies have found that memory-based dietary assessments underreport energy intake using doubly labeled water [7-9]. For instance, older healthy adults were found to underreport energy intake using food diaries regardless of ethnicity [7,8]. Adults tend to underreport total carbohydrates [10], protein [11], specifically meat and dairy products [12], and snack foods [10]. Less is known about what types of foods within each macronutrient group are most likely to be misreported [13], with logical assumptions that foods with a negative health image (e.g., sweets) may be underreported and foods with a positive health image (e.g., fruits and vegetables) are more likely to be overreported [3]. Understanding specific foods that are typically misreported can be translated into the clinic to design assessment tools (e.g., food props) and methods (e.g., multi-pass questions) to facilitate accurate dietary assessment.

In controlled feeding studies, participants consume only foods and drinks that are prepared in the metabolic kitchen for an acute period. Although controlled feeding studies are labor-intensive and costly, they provide an opportunity to assess inconsistencies in memory-based dietary assessments against known provided meals [14]. The application of a 24 h recall to a controlled feeding study is not well understood, with several studies suggesting underreporting of macronutrients [15-18]. For example, a controlled feeding study in adults (n = 59) for twelve days demonstrated that subjects underreported energy intake by 5–21% using doubly labeled water [15]. Controlled feeding studies are analogous to eating meals outside the home, as participants have limited knowledge on how foods were prepared. Today, over a third of daily calories are consumed from foods prepared outside the home [19] and meals outside the home are associated with a higher total energy and fat intake [20]. Therefore, utilizing a controlled feeding study to assess the accuracy of memory-based nutritional assessments may elucidate shortcomings in nutritional assessment of premade meals.

The primary objective of this pilot study is to identify discrepancies in energy and macronutrient intake comparing self-reported intake assessed via 24 h dietary recall to food provided within a controlled feeding intervention. The secondary objective of this pilot study is to identify specific food groups that are typically misreported.

## 2. Materials and Methods

**Figure 1.**
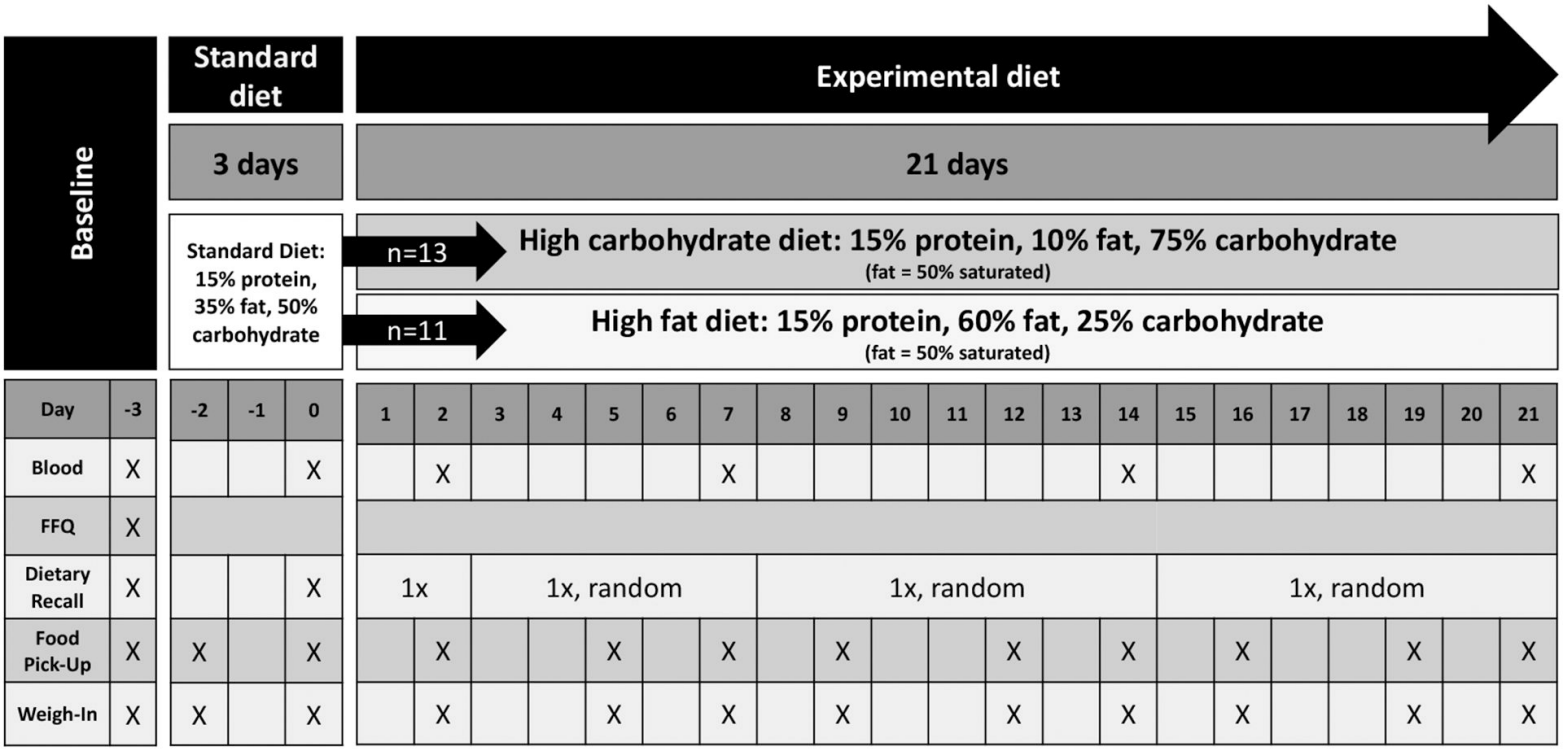
Metabolomic Analysis of Diet (MEAL) Study Design. All participants completed a standard diet followed by either a high carbohydrate or a high fat diet. Dietary recalls were collected up to six times throughout the study.

This report is a sub-analysis of a controlled feeding pilot study with a parallel randomized block design, called the Metabolomic Analysis of Diet (MEAL) study. The study was designed to test the metabolite response to two dietary interventions: a high-carbohydrate (HC) and a high-fat (HF) diet (Figure 1). Subjects were recruited using the University of Michigan research online portal (https://umhealthresearch.org, accessed on 1 July 2016). Participants included undergraduate and graduate students as well as community volunteers from the immediate vicinity. Inclusion criteria were age between 19 and 45 years, no history of metabolic disorders, body mass index (BMI) between 18.5 and 27, not currently taking metabolism-altering drugs and stable weight ±2 kg for the last 6 months. Exclusion criteria included food allergies, refusal to eat the food provided, need for special food considerations such as vegan, vegetarian, or religious food requirements, and regular smokers who had not stopped within the previous 6 months.

Participants included 12 males (7 HC, 5 HF) and 12 females (6 HC, 6 HF). Study visits occurred at the University of Michigan. Participants were randomly allocated to study intervention using a random number generator in Microsoft Excel. Randomization, enrollment, and assignment to dietary interventions were performed by the study team. The study team was not blinded to the assigned dietary intervention. Participants were blinded to their dietary intervention; however, it was easy to infer if they were on a HF or HC diet based on the foods given to them. The protocol is available on Deep Blue Repositories, through the University of Michigan library. This study was conducted between July 2016 and April 2017. The University of Michigan Medical School Institutional Review Board approved the study protocol and all participants provided written informed consent (HUM #000110543).

### Baseline Visit.

An initial study visit occurred with a Registered Dietitian (RD), a study coordinator, and a Registered Nurse (RN) or Licensed Practical Nurse (LPN) for an assessment of estimated energy intake, using the Institute of Medicine formulas [21]. Baseline body weight was collected in light clothing and stocking feet using a scale calibrated to the nearest 0.1 kg (Scale-Tronix Model 6002, White Plains, NY, USA). Baseline height was collected using a wall-mounted stadiometer in duplicate to the nearest 0.5 cm (Easy-Glide Bearing Stadiometer, Perspective Enterprises, Portage, MI, USA). The study team monitored participants’ weight at each food pick-up visit. If body weight changed by ≥2.5%, total calories of experimental diets were modified to return to initial body weight. The proportion of macronutrients within each experimental diet remained constant.

### Feeding Intervention.

For 24 days, all meals and drinks were provided for the participants. Meals were created at the metabolic kitchen at the University of Michigan and were picked up by participants twice per week. All participants consumed a standard (STD) diet for 3 days (15% protein, 35% fat, 50% carbohydrate) prior to their random assignment to one of the two experimental diet groups for 21 days; the HF diet (15% protein, 60% fat with 50% SFA, 25% carbohydrate) or the HC diet (15% protein, 10% fat with 50% SFA, 75% carbohydrate). All dietary interventions were eucaloric. Participants were asked to consume all provided food and return any uneaten food to the lab on subsequent visits to be subtracted from total intake. However, very few participants abided by this request due to leftover food spoilage in the cooler and the inconvenience of saving uneaten foods. The study team made all possible accommodations for varied food preferences, sensitivities, and requirements for all subjects. Sample dietary menus are provided in Supplemental Table S1. A four-day rotating menu was utilized.

### Dietary Assessment.

During the baseline visit, participants received training on using measuring instruments (e.g., cups and spoon) and food props (e.g., deck of cards is equivalent to 3 oz of meat) during subsequent 24 h dietary recalls. All participants had access to a written copy of a food-amount-reporting booklet (adapted from [22]), which included scalable pictures of bowls, glasses, and mugs with fullness markings and pictures of different cuts of meat, chicken, and fish. The Nutrition Data System for Research (NDSR) software (version 2015) was used for dietary assessment (Nutrition Coordinating Center, University of Minnesota, Minneapolis, MN, USA). All study RDs were certified in the collection of 24 h dietary recalls using NDSR, utilizing the multi-pass method for accurate assessment [23]. Up to six 24 h dietary recalls were conducted by study RDs, including at baseline (Day −3), during the standard diet (Day 0), and during the experimental diet (Day 2 and three other random times) (Figure 1). NDSR was used to extract provided and self-reported calories, macronutrients, and food groups.

### Statistical Analyses.

Cohort demographics were compared between the HC and HF experimental groups (unpaired Student’s *t*-test). Self-reported intake from the standard diet was quantified with a single 24 h dietary recall. Self-reported intake of calories and macronutrients from both experimental diets was quantified using an average across the 24 h dietary recalls. Self-reported intake of food groups from both experimental diets was analyzed separately for each 24 h dietary recall to reflect differences in the types of foods from the rotating experimental menu. Reported macronutrient intake was adjusted for total energy intake, calculating the percent calories from each macronutrient. Food groups were reported in number of servings. Percent difference between provided menu and self-reported servings of food groups was quantified using the equation (reported – provided)/[(reported + provided)/2] × 100. All statistical analyses comparing provided menu items and self-reported intake utilized paired Student’s *t*-tests. Statistical significance was described using a *p* < 0.05. All data analyses were performed using R version 4.0 [24].

## 3. Results

### 3.1. Description of Dietary Comparisons

Participants were 24 years old (19–32) on average, with a mean BMI of 22.8 kg/m^2^ (18.3–26.2). The BMI range spanned underweight (BMI < 18.5, n = 1), normal weight (18.5 ≤ BMI < 25, n = 17), and overweight (25 ≤ BMI < 30, n = 6). There were no significant differences in baseline weight, age, or BMI between the HC and HF experimental groups (Supplemental Table S2). Twenty-four participants (100%) completed the study. A 24 h dietary recall was completed by 14/24 participants during the STD diet. During the HC diet, participants completed one (one participant), two (two participants), or three (nine participants) 24 h dietary recalls. During the HF diet, participants completed one (one participant), two (two participants), three (eight participants), or four (one participant) 24 h dietary recalls.

### 3.2. Discrepancies in Energy and Macronutrient Intake between Dietary Recall vs. Provided Meals

**Figure 2.**
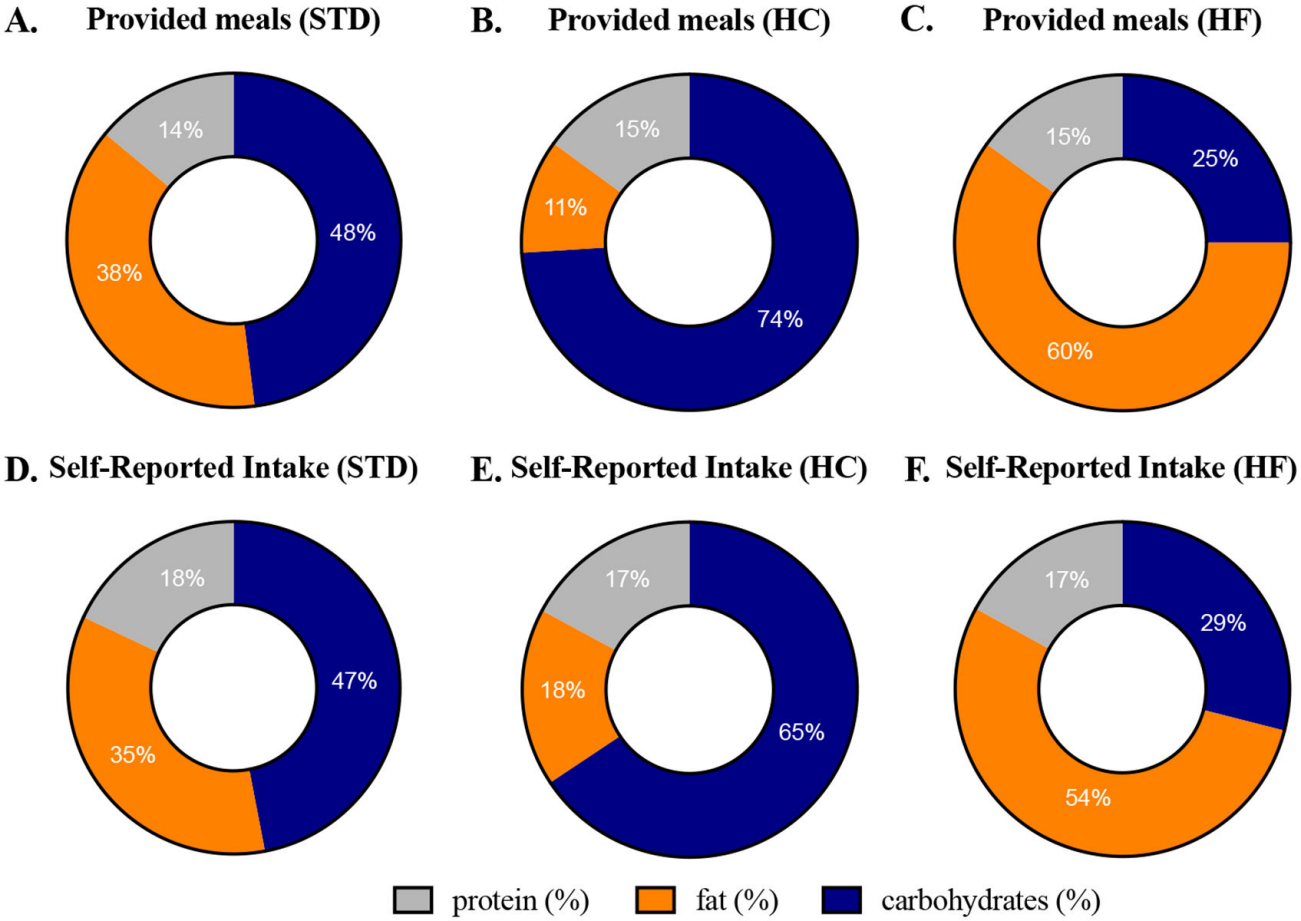
Macronutrient intake expressed as a percentage of total energy intake for provided meals during (**A**) standard (STD), (**B**) high-carbohydrate (HC), and (**C**) high-fat (HF) diets and self-reported intake during (**D**) standard, (**E**) high-carbohydrate, and (**F**) high-fat diets.

No caloric intake differences were observed between self-reported vs. provided meals in either the STD, HC, or HF diets (Table 1). Small differences (grams) of macronutrients in the diets were observed between self-reported intake vs. provided meals (Table 1). In the STD diet, participants reported a higher amount of protein than provided (123 g reported vs. 95 g provided, *p* = 0.03), specifically overreporting animal protein (86 g reported vs. 63 g provided, *p* = 0.04). In the HC diet, participants reported a higher amount of fat than provided (55 g reported vs. 34 g provided, *p* = 0.003). To extrapolate on these results, we compared differences in the type of fat—saturated, monounsaturated, and polyunsaturated—between self-reported intake vs. provided meals (Supplemental Table S3). In the HC diet, grams of saturated, monounsaturated, and polyunsaturated fat were significantly overreported. Macronutrients were adjusted for total energy intake using daily calories, as the amount of food each participant was provided varied by their weight, age, and sex per the Institute of Medicine equations. Adjusting for total energy intake demonstrated multiple discrepancies in provided vs. self-reported diets (Figure 2). In the STD diet, participants reported a higher percent of protein than provided (18% reported vs. 14% provided, *p* = 0.0004), with no observed differences in the percentage of carbohydrates and fat reported vs. provided. In the HC diet, participants reported a higher percent of protein than provided (17% reported vs. 15% provided, *p* = 6.1 × 10^−4^), a higher percent of fat than provided (18% reported vs. 11% provided, *p* = 8.5 × 10^−9^), and a lower percent of carbohydrates than provided (65% reported vs. 74% provided, *p* = 7.4 × 10^−9^). In the HF diet, participants reported a higher percent of protein than provided (17% reported vs. 15% provided, *p* = 0.03), a lower percent of fat than provided (54% reported vs. 60% provided, *p* = 3.7 × 10^−5^), and a higher percent of carbohydrates than provided (29% reported vs. 25% provided, *p* = 0.01). The type of fat was adjusted for total energy intake (Supplemental Table S4). In the HC diet, percent caloric intake of saturated, monounsaturated, and polyunsaturated fat was significantly overreported. In the HF diet, percent caloric intake of saturated fat was the only significantly underreported type of fat (23% reported vs. 28% provided, *p* = 0.0002).

### 3.3. Discrepancies in Servings of Food Groups between Dietary Recall vs. Provided Meals

**Figure 3.**
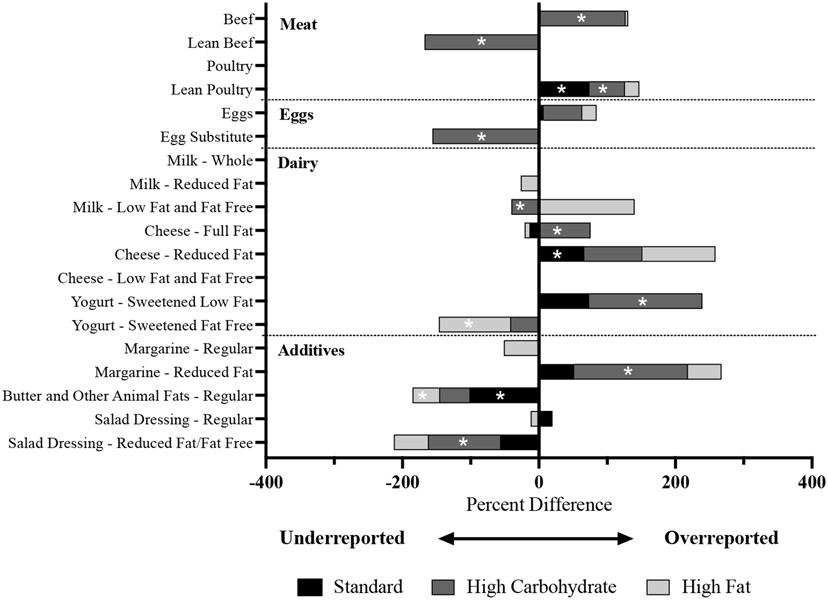
Percent difference in reporting meat, eggs, dairy, and additives stratified by dietary intervention. Percent difference reported as an additive effect by stacking bars for each dietary intervention. Significant differences between self-reported vs. provided servings of the food groups denoted with a white “*” (*p*-value < 0.05).

Foods were classified into servings of food groups in NDSR and self-reported vs. provided servings of the food groups were compared for each dietary intervention (Supplemental Table S5). Percent difference in reporting meats, eggs, milk products, and additives are represented in Figure 3. Several differences were observed comparing the self-reported vs. provided number of servings in the STD diet, with an overreporting of lean poultry (*p* = 0.001), an overreporting of reduced-fat cheese (*p* = 0.05), and an underreporting of butter (*p* = 0.0003). Many differences were observed comparing the self-reported vs. provided number of servings in the HC diet, with discrepancies in reporting whole- vs. refined-grain breads, regular vs. lean beef, and regular vs. reduced-fat salad dressings. Overall, individuals in the HC diet overreported beef (*p* = 0.0002), lean poultry (*p* = 0.034), full-fat cheese (*p* = 0.044), yogurt (*p* = 0.005), and reduced-fat margarine (*p* = 0.001). Several differences were observed between the self-reported vs. provided number of servings in the HF diet, including overreporting pasta (*p* = 0.012), overreporting yogurt (*p* = 0.05), and underreporting butter (*p* = 0.001). There were very few discrepancies between the self-reported vs. provided number of servings of fruits and vegetables across each dietary intervention.

## 4. Discussion

Dietary assessment methods based on self-report are prone to systematic and random errors [1,2]. The objective of this study was to compare self-reported dietary intake using 24 h recalls to provided menu items in a controlled feeding intervention. Self-reported energy-adjusted protein intake was greater than provided protein in each of the diets (Figure 2). This was specifically attributed to overreporting of animal protein, including beef and poultry (Figure 3), demonstrating the challenges in quantifying the size of a serving of meat. To our knowledge, three previous studies have measured differences in self-reported vs. actual intake of animal protein, with two studies finding underreporting [12,25] and one study finding no differences [26]. Additionally, we observed discrepancies in reporting fat content in meats (e.g., regular vs. lean beef in the HC diet), which may have contributed to the increased self-reported intake of saturated fat in the HC diet (Supplemental Table S3). The NDSR food prop booklet that participants used for this study includes several depictions of steaks, poultry pieces, and filets; however, many animal protein foods that people eat are in the form of casseroles, dishes, and stews, which can make the amount and the type of fat within the protein source challenging to quantify. Recent attempts to increase the accuracy of dietary recalls include using digital images of foods (e.g., Automated Self-Administered 24-h [ASA-24] Dietary Assessment Tool) [27], increasing the number of multi-passes (e.g., Automated Multiple-Pass Method [AMPM]) [28], and text-based descriptions of portion sizes [29]. Understanding and addressing the limitations in the quantification of animal protein within dietary recalls is important as many epidemiologic studies draw conclusions about animal protein- and nutrition-related chronic conditions [30].

All the meals within this controlled feeding study were premade and included lasagna, cream of broccoli soup, and breakfast sandwiches. Therefore, it can be considered that our study is analogous to eating premade foods outside of the home (e.g., restaurants and takeout), in which foods are often misreported due to preparation differences. Importantly, our experimental diets were designed to be excessively high in fat or carbohydrates to support our primary aim of assessing the metabolite response to a high-fat or high-carbohydrate acute interventions (manuscript pending). Underreporting percent calories from fat in the HF diet and underreporting of percent calories from carbohydrates in the HC diet may be attributed to this design, in which the experimental diets were exaggerated from a typical dietary pattern. For instance, the HF diet utilized “hidden” fats in dairy products (e.g., half and half and butter), dishes (e.g., cheesy potato bisque), and meats (e.g., sausage links), which may have contributed to the underreporting of caloric intake of saturated fat in the HF diet (Supplemental Table S4).

Our study is relevant to current eating patterns, as the consumption of food prepared outside of the home has increased through the years [19]. In adults in 2011–2012, premade food eaten outside of the home contributed to 34% of daily caloric intake, growing from 17% in 1977–78 [19], demonstrating the necessity of creating tools to help measure nutritional information in premade meals. It is postulated that with advances in artificial intelligence, digital technology (e.g., mobile applications and wearable technologies) can provide real-time collection of dietary data without the need for self-reports [31]. For instance, researchers have developed wearable cameras that capture food images with sensors to monitor meal intake (e.g., Automatic Ingestion Monitory 2 [AIM-2]) [32]. The assessment of dietary intake by wearable sensors and application is a strong area of research; however, it is necessary to increase accessibility and technical assistance for these technologies prior to translating them into the clinic [33]. Furthermore, current artificial intelligence technologies may not adequately quantify high-caloric food additives in meals, such as butter.

The strengths of our study include its longitudinal experimental design with the comparison of three different dietary interventions with varying macronutrient composition. Multiple 24 h recalls were collected from participants during the experimental diet by trained Registered Dietitians using the multi-pass methods in NDSR; however, most participants were not able to be contacted for all four 24 h recalls in the experimental diet. Analyses compared self-reported intakes to provided meals; however, we cannot be certain if the participants ate all the provided foods, as a limitation of this study was that most participants did not return uneaten foods to the clinical research unit due to its inconvenience and high levels of food spoilage. Our study included an equal distribution of males and females; however, we only recruited a small sample size of “healthy” young adults, limiting the generalizability of our study. Of interest, previous studies have demonstrated that individuals with obesity underreport their dietary intake [34]. In a sub-analysis, we did not observe any significant associations between BMI and misreporting dietary intake, potentially due to the small range of BMI in the cohort; however, a non-significant trend was observed that individuals with a higher BMI underreported calories in the HF diet (*p* = 0.18). Future work on this study question should consider a wider range of BMIs spanning several weight status categories. Our main objective for the MEAL study is to classify metabolite biomarkers of high fat and high carbohydrate intake; therefore, we created the menus with specific food adaptions to exaggerate either fat or carbohydrate intake that may have been unknown to participants (e.g., regular vs. low-fat ingredients). This may have increased misreporting of specific food groups (e.g., regular vs. lean beef); however, certain trends were observed across multiple diets (e.g., overreporting poultry). Further, subjects were not screened for binge eating disorders or other psychosocial conditions which may contribute to inaccuracy known to affect dietary recall [35].

## 5. Conclusions

Our results provide preliminary data suggesting that specific protein-rich food groups, including poultry and beef, tend to be overreported in a 24 h dietary recall. Furthermore, we observed that individuals on a high-macronutrient diet tend to underreport their most abundant macronutrient. Future directions include expanding this preliminary study to test additional dietary interventions with a range of macronutrients compositions. Future work should consider comparing self-reported 24 h recalls and artificial intelligence sensors in a controlled feeding study, utilizing specifically targeted mobile applications that are freely available to the general population. Future work should consider recruiting case vs. control patients, targeting disease states in which nutritional analysis has high implications. Studies with larger and more diverse study populations should be conducted to strengthen and extend the generalizability of these results.

## Supplementary Material

Supplemental Table S1

Supplemental Table S2

Supplemental Table S3

Supplemental Table S4

Supplemental Table S5

## Figures and Tables

**Table 1.** Absolute quantities of calories and macronutrients, stratified by the type of diet. Values are mean ± standard deviation. *p*-value < 0.05 is bolded.

	Provided Intake	Reported Intake	*p*-Value
standard diet			
calories (kcal)	2676 ± 498	2726 ± 668	0.82
carbohydrates (g)	328 ± 70	322 ± 88	0.86
fat (g)	115 ± 35	109 ± 35	0.68
protein (g)	95 ±19	123 ± 41	**0.03**
high-carbohydrate diet			
calories (kcal)	2777 ± 495	2823 ± 991	0.88
carbohydrates (g)	535 ± 97	479 ± 171	0.32
fat (g)	34 ± 9	55 ± 21	**0.003**
protein (g)	103 ± 19	119 ± 41	0.23
high-fat diet			
calories (kcal)	2722 ± 496	2791 ± 565	0.77
carbohydrates (g)	173 ± 33	205 ± 55	0.12
fat (g)	185 ± 34	171 ± 37	0.36
protein (g)	101 ± 18	116 ± 28	0.15

## Data Availability

Data described in the manuscript, code book, and analytic code will be made available upon request.

## References

[1] Archer E; Marlow ML; Lavie CJ Controversy and debate: Memory-Based Methods Paper 1: The fatal flaws of food frequency questionnaires and other memory-based dietary assessment methods. J. Clin. Epidemiol 2018, 104, 113–124. 30121379 10.1016/j.jclinepi.2018.08.003

[2] Archer E; Pavela G; Lavie CJ The Inadmissibility of What We Eat in America and NHANES Dietary Data in Nutrition and Obesity Research and the Scientific Formulation of National Dietary Guidelines. Mayo Clin. Proc 2015, 90, 911–926. 26071068 10.1016/j.mayocp.2015.04.009 PMC4527547

[3] Macdiarmid J; Blundell J Assessing dietary intake: Who, what and why of under-reporting. Nutr. Res. Rev 1998, 11, 231–253. 19094249 10.1079/NRR19980017

[4] Chi SA; Lee H; Lee JE; Lee HS; Kim K; Yeo IK An ensemble method based on marginal-effect models (EMM) for estimating usual food intake from single-day dietary data and internal/external two-day dietary data. Eur. J. Clin. Nutr 2023, 77, 325–334. 36357566 10.1038/s41430-022-01231-1

[5] Palaniappan U; Cue RI; Payette H; Gray-Donald K Implications of day-to-day variability on measurements of usual food and nutrient intakes. J. Nutr 2003, 133, 232–235. 12514296 10.1093/jn/133.1.232

[6] Young LR; Nestle MS Portion sizes in dietary assessment: Issues and policy implications. Nutr. Rev 1995, 53, 149–158. 7478309 10.1111/j.1753-4887.1995.tb01542.x

[7] Tomoyasu NJ; Toth MJ; Poehlman ET Misreporting of total energy intake in older men and women. J. Am. Geriatr. Soc 1999, 47, 710–715. 10366171 10.1111/j.1532-5415.1999.tb01594.x

[8] Tomoyasu NJ; Toth MJ; Poehlman ET Misreporting of total energy intake in older African Americans. Int. J. Obes. Relat. Metab. Disord 2000, 24, 20–26. 10702746 10.1038/sj.ijo.0801079

[9] Taren DL; Tobar M; Hill A; Howell W; Shisslak C; Bell I; Ritenbaugh C The association of energy intake bias with psychological scores of women. Eur. J. Clin. Nutr 1999, 53, 570–578. 10452412 10.1038/sj.ejcn.1600791

[10] Poppitt SD; Swann D; Black AE; Prentice AM Assessment of selective under-reporting of food intake by both obese and non-obese women in a metabolic facility. Int. J. Obes. Relat. Metab. Disord 1998, 22, 303–311. 9578234 10.1038/sj.ijo.0800584

[11] Heitmann BL; Lissner L; Osler M Do we eat less fat, or just report so? Int. J. Obes. Relat. Metab. Disord 2000, 24, 435–442. 10805500 10.1038/sj.ijo.0801176

[12] Garden L; Clark H; Whybrow S; Stubbs RJ Is misreporting of dietary intake by weighed food records or 24-h recalls food specific? Eur. J. Clin. Nutr. 2018, 72, 1026–1034. 29789710 10.1038/s41430-018-0199-6

[13] Gemming L; Ni Mhurchu C Dietary under-reporting: What foods and which meals are typically under-reported? Eur. J. Clin. Nutr. 2016, 70, 640–641. 26669571 10.1038/ejcn.2015.204

[14] Most MM; Ershow AG; Clevidence BA An overview of methodologies, proficiencies, and training resources for controlled feeding studies. J. Am. Diet. Assoc 2003, 103, 729–735. 12778045 10.1053/jada.2003.50132

[15] Stubbs RJ; O’Reilly LM; Whybrow S; Fuller Z; Johnstone AM; Livingstone MB; Ritz P; Horgan GW Measuring the difference between actual and reported food intakes in the context of energy balance under laboratory conditions. Br. J. Nutr 2014, 111, 2032–2043. 24635904 10.1017/S0007114514000154

[16] Whybrow S; Stubbs RJ; Johnstone AM; O’Reilly LM; Fuller Z; Livingstone MB; Horgan GW Plausible self-reported dietary intakes in a residential facility are not necessarily reliable. Eur. J. Clin. Nutr 2016, 70, 130–135. 26220569 10.1038/ejcn.2015.124

[17] Schebendach JE; Porter KJ; Wolper C; Walsh BT; Mayer LE Accuracy of self-reported energy intake in weight-restored patients with anorexia nervosa compared with obese and normal weight individuals. Int. J. Eat. Disord 2012, 45, 570–574. 22271488 10.1002/eat.20973 PMC4469285

[18] Westerterp KR; Goris AH Validity of the assessment of dietary intake: Problems of misreporting. Curr. Opin. Clin. Nutr. Metab. Care 2002, 5, 489–493. 12172471 10.1097/00075197-200209000-00006

[19] Saksena MJ; Okrent AM; Anekwe TD; Cho C; Dicken C; Effland A; Elitzak H; Guthrie J; Hamrick KS; Hyman J; America’s Eating Habits: Food Away from Home; United States Department of Agriculture, Economic Research Service: Washington, DC, USA, 2018.

[20] Lachat C; Nago E; Verstraeten R; Roberfroid D; Van Camp J; Kolsteren P Eating out of home and its association with dietary intake: A systematic review of the evidence. Obes. Rev 2012, 13, 329–346. 22106948 10.1111/j.1467-789X.2011.00953.x

[21] Institute of Medicine (IOM) of the National Academies. Dietary Reference Intakes: Research Synthesis Workshop Summary; The National Academies Press: Washington, DC, USA, 2007.

[22] van Horn LV; Stumbo P; Moag-Stahlberg A; Obarzanek E; Hartmuller VW; Farris RP; Kimm SY; Frederick M; Snetselaar L; Liu K The Dietary Intervention Study in Children (DISC): Dietary assessment methods for 8- to 10-year-olds. J. Am. Diet. Assoc 1993, 93, 1396–1403. 8245373 10.1016/0002-8223(93)92241-o

[23] Feskanich D; Sielaff BH; Chong K; Buzzard IM Computerized collection and analysis of dietary intake information. Comput. Methods Programs Biomed 1989, 30, 47–57. 2582746 10.1016/0169-2607(89)90122-3

[24] R Core Team. R: A Language and Environment for Statistical Computing; R Foundation for Statistical Computing: Vienna, Austria, 2021.

[25] Stewart C; Bianchi F; Frie K; Jebb SA Comparison of Three Dietary Assessment Methods to Estimate Meat Intake as Part of a Meat Reduction Intervention among Adults in the UK. Nutrients 2022, 14, 411. 35276771 10.3390/nu14030411 PMC8839883

[26] McNaughton SA; Mishra GD; Bramwell G; Paul AA; Wadsworth ME Comparability of dietary patterns assessed by multiple dietary assessment methods: Results from the 1946 British Birth Cohort. Eur. J. Clin. Nutr 2005, 59, 341–352. 15523484 10.1038/sj.ejcn.1602079

[27] Kirkpatrick SI; Potischman N; Dodd KW; Douglass D; Zimmerman TP; Kahle LL; Thompson FE; George SM; Subar AF The Use of Digital Images in 24-Hour Recalls May Lead to Less Misestimation of Portion Size Compared with Traditional Interviewer-Administered Recalls. J. Nutr 2016, 146, 2567–2573. 27807039 10.3945/jn.116.237271 PMC5118765

[28] Moshfegh AJ; Rhodes DG; Baer DJ; Murayi T; Clemens JC; Rumpler WV; Paul DR; Sebastian RS; Kuczynski KJ; Ingwersen LA; The US Department of Agriculture Automated Multiple-Pass Method reduces bias in the collection of energy intakes. Am. J. Clin. Nutr 2008, 88, 324–332. 18689367 10.1093/ajcn/88.2.324

[29] Lucassen DA; Willemsen RF; Geelen A; Brouwer-Brolsma EM; Feskens EJM The accuracy of portion size estimation using food images and textual descriptions of portion sizes: An evaluation study. J. Hum. Nutr. Diet 2021, 34, 945–952. 33761165 10.1111/jhn.12878 PMC9291996

[30] Naghshi S; Sadeghi O; Willett WC; Esmaillzadeh A Dietary intake of total, animal, and plant proteins and risk of all cause, cardiovascular, and cancer mortality: Systematic review and dose-response meta-analysis of prospective cohort studies. BMJ 2020, 370, m2412. 32699048 10.1136/bmj.m2412 PMC7374797

[31] Limketkai BN; Mauldin K; Manitius N; Jalilian L; Salonen BR The Age of Artificial Intelligence: Use of Digital Technology in Clinical Nutrition. Curr. Surg. Rep 2021, 9, 20. 34123579 10.1007/s40137-021-00297-3 PMC8186363

[32] Doulah A; Ghosh T; Hossain D; Marden T; Parton JM; Higgins JA; McCrory MA; Sazonov E Energy intake estimation using a novel wearable sensor and food images in a laboratory (pseudo-free-living) meal setting: Quantification and contribution of sources of error. Int. J. Obes 2022, 46, 2050–2057. 10.1038/s41366-022-01225-w PMC12938214 36192533

[33] Das SK; Miki AJ; Blanchard CM; Sazonov E; Gilhooly CH; Dey S; Wolk CB; Khoo CSH; Hill JO; Shook RP Perspective: Opportunities and Challenges of Technology Tools in Dietary and Activity Assessment: Bridging Stakeholder Viewpoints. Adv. Nutr 2022, 13, 1–15. 34545392 10.1093/advances/nmab103 PMC8803491

[34] Wehling H; Lusher J People with a body mass index ≥30 under-report their dietary intake: A systematic review. J. Health Psychol 2019, 24, 2042–2059. 28810493 10.1177/1359105317714318

[35] Bartholome LT; Peterson RE; Raatz SK; Raymond NC A comparison of the accuracy of self-reported intake with measured intake of a laboratory overeating episode in overweight and obese women with and without binge eating disorder. Eur. J. Nutr 2013, 52, 193–202. 22302613 10.1007/s00394-012-0302-z PMC4056663

