# Supplementary material for: Comparing Self-Reported Dietary Intake to Provided Diet during a Randomized Controlled Feeding Intervention: A Pilot Study
Source: Dietetics (Basel). Author manuscript; Available in PMC 2023 Dec 15. (PMC10722558; doi:10.3390/dietetics2040024)
Supplement: Supplemental Table S1 [file NIHMS1950702-supplement-Supplemental_Table_S1.pdf]

Supplemental Table S1. Samples menus of food provided to participants. Sample menus based on a 2000 kcal diet for the standard diet (A-C), high carbohydrate diet (D-G), and high fat diet (H-K). Food, amount, calories, and grams of protein, fat, saturated fat, and carbohydrates are reported. Participants may drink non-caloric beverages of their choice in each of the diets.

A) Sample Standard Menu #1

FOOD	AMOUNT	CALORIES	PROTEIN (g)	FAT (g)	SATURATED FAT (g)	CARB (g)
**Breakfast**						
Rice Chex	1 bowl	70	1	0	0	16
2% milk	240 mL	113	7.6	4.4	2.7	11
Strawberry yogurt	6 oz. carton	160	5	1.5	0	33
Banana	1 medium	111	1.2	0.6	0	28
**Lunch**						
Pepperoni pizza	1 unit	381	19.1	14.1	7.5	42
Applesauce	1 container	51	0.2	0.1	0	13
Trail mix	1 package	290	8	18	4.5	27
**Dinner**						
Chicken primavera	325 g	346	29.4	8.5	3.3	38
Butter in primavera	10 g	72	0	8	5.2	0
Cream of broccoli soup	168 g	137	4.8	7.3	2.3	14
Butter in soup	10 g	72	0	8	5.2	0
Chocolate chip cookie	1 unit (36 g)	160	2	8	4	23
**Summary**						
Provided		1963	78	79	35	245
Percentage of calories			16%	36%	16%	50%
Goal		2000	76	78	39	250
Percentage of calories			15%	35%	18%	50%

B) Sample Standard Menu #2

FOOD	AMOUNT	CALORIES	PROTEIN (g)	FAT (g)	SATURATED FAT (g)	CARB (g)
**Breakfast**						
Breakfast sandwich	1	251	19.6	7.9	4.5	25
Orange juice	2 cartons	122	0	0	0	28
Clementine	1	50	0.7	0.2	0	13
Raisins	30 g	94	0.8	0	0	25
**Lunch**						
Sliced turkey	40 g	48	12	0	0	0
Mayonnaise-light	2 packets	50	0	5	0	0
Tomato	3 slices	12	0.5	0.2	0	3
Potato chips	1 bag (28 g)	160	2	11	3	15
String cheese	2 sticks	160	12	12	7	0
White bread	1 slice	83	2.5	0.5	0	16
Lemonheads	2 boxes	180	0	0	0	44
**Dinner**						
Macaroni and cheese	120 g	231	10	13.8	7.8	16
BBQ chicken breast	60 g	75	10	2.1	0.2	4
Garden medley vegetables	112 g	42	1.7	0	0	8
Butter in macaroni and cheese and vegetables	5 g	36	0	4	2.6	0
Donut chocolate	1 unit	365	3.3	20.7	8.4	42
**Summary**						
Provided		2042	78	78	33	255
Percentage of calories			15%	34%	15%	50%
Goal		2000	76	78	39	250
Percentage of calories			15%	35%	18%	50%

C) Sample Standard Menu #3

FOOD	AMOUNT	CALORIES	PROTEIN (g)	FAT (g)	SATURATED FAT (g)	CARB (g)
**Breakfast**						
Pancakes	2	126	2.8	1.1	0.4	26
Pancake syrup	1 packet	120	0	0	0	30
Butter on pancakes	10 g	72	0	8	5.2	0
Orange juice	1 carton	61	0	0	0	14
Omelet with cheese	1 package	288	20.2	21	7.3	6
**Lunch**						
Garden burger	1 patty	180	5.1	9.5	1	21
White bun	1 unit	110	3	2	0	21
Mayonnaise-light	2 packets	50	0	5	0	0
Cheddar cheese	23 g	84	5.3	7.3	4.6	0
Potato chips	1 bag (28 g)	160	2	11	3	15
Banana	medium	111	1.2	0.6	0	28
**Dinner**						
Chicken parmesan	157 g	233	27.6	7.7	1.9	13
Penne	98 g	159	5.3	0.8	0	32
Marinara sauce	56 g	24	1	0.4	0	4
White roll	28 g	80	2.5	1	0.5	15
Butter for roll	5 g	36	0	4	2.6	0
Raspberry sherbet	1 carton	120	0	1.5	1	27
**Summary**						
Provided		2014	76	81	28	252
Percentage of calories			15%	36%	12%	50%
Goal		2000	76	78	39	250
Percentage of calories			15%	35%	18%	50%

D) Sample High Carbohydrate Menu #1

FOOD	AMOUNT	CALORIES	PROTEIN (g)	FAT (g)	SATURATED FAT (g)	CARB (g)
**Breakfast**						
Frosted Flakes	1 bowl	110	1	0	0	26
Skim milk	236 g carton	79	7.7	0.4	0.3	11
Fat free strawberry yogurt	170 g carton	90	6	0	0	17
Apple juice	4 oz carton	61	0	0	0	15
**Lunch**						
Chicken noodle soup	206 g	50	3	0	0.5	8
Fresh cut vegetables	96 g	21	0.7	0.3	0	5
Ranch dressing	30 g	44	0.8	3.4	1	2.8
Grilled chicken breast	67 g	85	15.5	2.4	0.2	0
Lettuce leaf	15 g	4	0	0	0	0.4
Wheat bun	1 unit	145	7	1	0	28
Butter on bun	5 g	36	0	4	2.6	0
Ginger ale	8 oz can	96	0	0	0	26
Banana	small	90	1.1	0.3	0.1	23
**Dinner**						
Lasagna	78 g	118	9	5.4	3	7
Grape juice	2 cartons	122	0	0	0	30
Tossed greens	116 g	16	1.2	0.2	0	3
Raisins in salad	32 g	90	0.9	0.1	0	24
Fat free Italian dressing	22 g	10	0	0	0	2
Garden vegetable medley	113 g	42	1.7	0	0	8
Lemon ice	23 g carton	80	0	0	0	20
Sliced peaches	140 g	66	0	0	0	14.9
**Snacks**						
Smoothie with banana, honey, coconut, juice, protein, and yogurt	240 g	243	9.6	5.1	3.6	43
Skim milk	236 g carton	79	7.7	0.4	0.3	11

Vanilla Carnation Instant Breakfast, no sugar	20 g packet	70	5	0	0	12
Unsalted crackers	2 packages	50	2	0	0	10
Grape jelly	3 packets	105	0	0	0	27
**Summary**						
Provided		2006	80	23	12	374
Percentage of calories			16%	11%	5%	75%
Goal		2000	77	22	11	375
Percentage of calories			15	10	5	75

E) Sample High Carbohydrate Menu #2

FOOD	AMOUNT	CALORIES	PROTEIN (g)	FAT (g)	SATURATED FAT (g)	CARB (g)
**Breakfast**						
Orange juice	1 carton	61	0	0	0	14
Oatmeal	142 g	92	2.7	1.8	0	17
Raisins	35 g	110	1	0.2	0	27
Brown sugar	18 g	68	0	0	0	17.7
Skim milk	8 oz carton	79	7.7	0.4	0.3	11
**Lunch**						
Tuna salad	68 g	88	13	3.5	0.4	1.7
Wheat bread	2 slices	146	8	0.6	0	28
Pretzels	28 g	110	2	1	0	23
Clementine	1	50	0.7	0.2	0	13
Lemon ice	23 g carton	80	0	0	0	20
Soft drink	8 oz can	90	0	0	0	24
**Dinner**						
Chili	160 g	137	8.5	3.8	1.5	16
Baked potato	250 g	247	5.2	0.2	0.1	57
Butter on potato	4 g	29	0	3.3	2.1	0
Sour cream	1/3 packet	20	0.3	1.7	1	0.7
Raspberry sherbet	1 carton	120	0	1.5	1	27
Skim milk	236 g carton	79	7.7	0.4	0.3	11
**Snacks**						
Smoothie with banana, honey, coconut, juice, protein, and yogurt	240 g	243	9.6	5.1	3.6	43
Skim milk	236 g carton	79	7.7	0.4	0.3	11
Vanilla Carnation Instant Breakfast, no sugar	20 g packet	70	5	0	0	12
**Summary**						
Provided		2002	79	24	11	375
Percentage of calories			16%	11%	5%	75%

Goal	2000	77	22	11	375
Percentage of calories		15%	10%	5%	75%

F) Sample High Carbohydrate Menu #3

FOOD	AMOUNT	CALORIES	PROTEIN (g)	FAT (g)	SATURATED FAT (g)	CARB (g)
**Breakfast**						
Raisin Bran	1 bowl	126	3	1	0	27
Extra raisins in cereal	32 g	90	0.9	0.1	0	24
Honey	2 packets	60	0	0	0	16
Skim milk	236 g carton	79	7.7	0.4	0.3	11
Clementine	1	50	0.7	0.2	0	13
Grape juice	1 carton	61	0	0	0	15
**Lunch**						
White bread bun	1 unit	114	3	2	0	21
Sliced ham	57 g	50	9	1.5	0.5	1
Sliced turkey	33 g	40	9.7	0	0	0
Leaf lettuce	15 g	4	0	0	0	0.4
American cheese	17 g	51	2.6	4.3	2.6	0.9
Hellmans light mayonnaise	6 g	13	0	1.4	0	0.2
Pretzels	28 g package	110	2	1	0	23
Grapes	120 g	83	0.9	0.2	0	21
Raspberry sherbet	1 carton	120	0	1.5	1	27
Skim milk	236 g carton	79	7.7	0.4	0.3	11
**Dinner**						
Penne pasta	99 g	160	5.3	0.8	0	32
Marinara sauce	113 g	48	2	0.8	0	8
Parmesan cheese	1 packet	15	1.3	1	0.6	0
Green beans	90 g	25	1.4	0.2	0	6
Tossed greens	116 g	16	1.2	0.2	0	3
Fat free Italian dressing	22 g	10	0	0	0	2
Orange jello	1 carton	73	1.5	0	0	17
Soft drink	1 can	95	0	0	0	24
Banana	small	90	1.1	0.3	0.1	23
Unsweetened coconut	5 g	33	0.3	3.2	2.9	1.2

**Snacks**						
Skim milk	236 g carton	79	7.7	0.4	0.3	11
Vanilla Carnation Instant Breakfast, no sugar	20 g packet	70	5	0	0	12
Chocolate pudding	1 carton	128	3	2	1.5	24
**Summary**						
Provided		1976	77	23	10	375
Percentage of calories			16%	10%	5%	76%
Goal		2000	77	22	11	375
Percentage of calories			15%	10%	5%	75%

G) Sample High Carbohydrate Menu #4

FOOD	AMOUNT	CALORIES	PROTEIN (g)	FAT (g)	SATURATED FAT (g)	CARB (g)
**Breakfast**						
Omelet half serving	120 g	50	4.4	3.4	1.1	0.4
Pancakes reduced sugar	2	126	2.8	1.1	0.4	26
Pancake syrup	1 package	120	0	0	0	30
Skim milk	236 g carton	79	7.7	0.4	0.3	11
Orange juice	1 carton	60	0	0	0	14
**Lunch**						
Cream of tomato soup	170 g bowl	113	4.6	3	1.8	17
White bread	1 slice	83	2.5	0.5	0	16
Sliced ham	22 g	19	3.5	0.6	0.2	0.4
Swiss cheese	15 g	56	3.6	4.6	3.1	0
Pretzels	28 g	110	2	1	0	23
Apple juice	8 oz	122	0	0	0	30
Yogurt Greek strawberry	140 g	121	11	0	0	20.5
Unsweetened coconut	5 g	33	0.3	3.2	2.9	1.2
**Dinner**						
BBQ sauce packet	2 packets	30	0	0	0	8
Grilled chicken breast	85 g	108	19.6	3	0.3	0
Wild rice half	50 g	48	1.1	0.8	0.1	9
White rice	55 g	63	1.3	0.2	0	18.5
Garden vegetable medley	113 g	40	1.7	0	0	8
Strawberry jello	116 g	71	1	0	0	17
Vernors ginger ale	8 oz can	100	0	0	0	26
Raisins	20 g	60	0.6	0	0	15
**Snacks**						
Skim milk	236 g carton	79	7.7	0.4	0.3	11
Rice Chex	1 bowl	70	1	0	0	16
Sugar for cereal	2 packets	22	0	0	0	6
Banana	small	90	1.1	0.3	0.1	23

Unsalted crackers	2 packages	50	2	0	0	10
Fruit jelly	2 packages	70	0	0	0	18
**Summary**						
Provided		1997	80	23	11	375
Percentage of calories			16%	10%	5%	75%
Goal		2000	77	22	11	375
Percentage of calories			15%	10%	5%	75%

H) Sample High Fat Menu #1

FOOD	AMOUNT	CALORIES	PROTEIN (g)	FAT (g)	SATURATED FAT (g)	CARB (g)
**Breakfast**						
Egg omelet with cheese	99 g	180	10	13	5	4
Sausage link	2 units	146	6	13.2	5.3	1
Wheat bread for toast	1 slice	73	4	0.3	0	14
Butter for bread	10 g	72	0	8	5.1	0
Half and half	30 g	39	0.9	3.5	2.2	1.3
**Lunch**						
Grilled ham and cheese	138 g	344	20.6	17.8	10	28
Potato chips	1 bag (28 g)	160	2	11	3	15
**Dinner**						
Meatloaf	125 g	213	15.8	11.2	4.3	12.3
Macaroni and cheese	95 g	180	7.8	10.8	6	12.5
Green beans	91 g	25	1.4	0.2	0	6
Butter on beans/macaroni and cheese	30 g	216	0.2	24.4	15.4	0
**Snacks**						
Cheesecake	97 g slice	291	4.7	19.7	12.5	26
**Summary**						
Provided		1943	74	133	69	120
Percentage of calories			15%	62%	32%	25%
Goal		2000	76	133	67	125
Percentage of calories			15%	60%	30%	25%

I) Sample High Fat Menu #2

FOOD	AMOUNT	CALORIES	PROTEIN (g)	FAT (g)	SATURATED FAT (g)	CARB (g)
**Breakfast**						
Half and half	30 g	39	0.9	3.5	2.2	1.3
Oatmeal	195 g	123	3.6	2.4	0	23
Coconut oil in oatmeal	11 g	95	0	11	9.5	0
Butter in oatmeal	10 g	72	0	8.1	5.1	0
No calorie sweetener	1 packet	0	0	0	0	0
Vanilla Greek yogurt	125 g	91	10.8	0	0	12.5
**Lunch**						
Caesar salad	100 g	49	5.1	2.9	1.8	2
Caesar dressing	1 packet	233	1	25	4	1
Grilled chicken breast	75 g	95	17.2	2.6	0.3	0
Corn	45 g	36	1.2	0.2	0.05	8.5
Butter on corn and on chicken	10 g	72	0	8	5.2	0
**Dinner**						
Potato crusted cod	85 g	151	15.8	6	2.3	7.5
Tartar sauce	1 packet	40	0	4	0	2
Baked potato (half)	112 g	113	2.3	0.1	0	26.1
Sour cream	1 packet	60	1	5	3	2
Broccoli	85 g	21	2.3	0.1	0	4
Shredded cheddar on vegetables	29 g	113	7.1	9.1	5.1	1
Butter on broccoli	10 g	72	0	8.1	5.1	0
Butter in potato	14 g	100	0.1	11.4	7.2	0
**Snacks**						
Almond joy	45 g	220	2	13	8	26
String cheese	28-29 g stick	80	6	6	3.5	0
Potato chips	1 bag (28 g)	160	2	11	3	15
**Summary**						
Provided		2035	78	138	65	132
Percentage of calories			15%	61%	29%	26%

Goal	2000	76	133	67	125
Percentage of calories		15%	60%	30%	25%

J) Sample High Fat Menu #3

FOOD	AMOUNT	CALORIES	PROTEIN (g)	FAT (g)	SATURATED FAT (g)	CARB (g)
**Breakfast**						
Breakfast wrap	1 (125 g)	328	16.6	16	6	29
Half and half	30 g	39	0.9	3.5	2.2	1.3
**Lunch**						
Grilled cheese	1 sandwich	343	16.3	19.2	11.1	28
Potato chips	1 bag (28 g)	160	2	11	3	15
**Dinner**						
Grilled chicken breast	72 g	91	16.7	2.6	0.3	0
Broccoli	82 g	23	2.5	0.1	0	4
Butter on broccoli	15 g	108	0.1	12.2	7.7	0
Garden vegetable medley	57 g	21	0.9	0	0	4
Butter in macaroni and cheese	15 g	108	0.1	12.2	7.7	0
Macaroni & cheese	196 g	375	16.2	22.4	12.6	26
Butter in vegetable	15 g	108	0.1	12.2	7.7	0
Vegetable oil in macaroni and cheese	10 g	91	0	10	0	0
**Snacks**						
Cheesecake	68 g	195	3.1	13.2	8.4	17
**Summary**						
Provided		1994	76	135	67	124
Percentage of calories			15%	61%	30%	25%
Goal		2000	76	133	67	125
Percentage of calories			15%	60%	30%	25%

K) Sample High Fat Menu #4

FOOD	AMOUNT	CALORIES	PROTEIN (g)	FAT (g)	SATURATED FAT (g)	CARB (g)
**Breakfast**						
Egg omelet with cheese	99 g	180	10	13	5	4
Sausage link	2 units	146	6	13.2	5.3	1
Half and half	30 g	39	0.9	3.5	2.2	1.3
**Lunch**						
Cheesy potato bisque	1 serving (170 g)	180	6.5	10.4	6.5	15
Butter in soup	10 g	72	0	8	5.1	0
Roast beef	81 g	110	10.3	4.9	1.9	4.5
Cheddar cheese	23 g	84	5.3	7.3	4.6	0
Wheat roll	1 (35 g)	81	3.1	0.8	0	15
Butter on roll	10 g	72	0	8	5.1	0
Mustard packet	one	3	0.2	0.2	0	0.3
**Dinner**						
Meat sauce	113.5 g	113	7.7	5.3	0	9
Penne plain	99 g	159	5.3	0.8	0	32
Butter in penne	20 g	144	0	16	10.2	0
Broccoli	82 g	23	2.5	0.1	0	0
Cheddar cheese on broccoli	23 g	84	5.5	7.3	4.6	0
Butter in broccoli	7 g	50	0	5.6	3.6	0
**Snacks**						
Chocolate cake	78 g	285	3	17.7	6.9	31
**Summary**						
Provided		2009	77	135	66	118
Percentage of calories			15%	61%	30%	24%
Goal		2000	76	133	67	125
Percentage of calories			15%	60%	30%	25%
