# Supplementary material for: Comparing Self-Reported Dietary Intake to Provided Diet during a Randomized Controlled Feeding Intervention: A Pilot Study
Source: Dietetics (Basel). Author manuscript; Available in PMC 2023 Dec 15. (PMC10722558; doi:10.3390/dietetics2040024)
Supplement: Supplemental Table S3 [file NIHMS1950702-supplement-Supplemental_Table_S3.pdf]

Supplemental Table S3. Absolute quantities of calories and macronutrients, stratified by the type of diet. Values are mean ± standard deviation. P-value < 0.05 is bolded.

	**Provided Intake**	**Reported Intake**	**p-value**
	**standard diet**		
calories (kcal)	2676 ± 498	2726 ± 668	0.82
carbohydrates (g)	328 ± 70	322 ± 88	0.86
fat (g)	115 ± 35	109 ± 35	0.68
protein (g)	95 ± 19	123 ± 41	**0.03**
	**high carbohydrate diet**		
calories (kcal)	2777 ± 495	2823 ± 991	0.88
carbohydrates (g)	535 ± 97	479 ± 171	0.32
fat (g)	34 ± 9	55 ± 21	**0.003**
protein (g)	103 ± 19	119 ± 41	0.23
	**high fat diet**		
calories (kcal)	2722 ± 496	2791 ± 565	0.77
carbohydrates (g)	173 ± 33	205 ± 55	0.12
fat (g)	185 ± 34	171 ± 37	0.36
protein (g)	101 ± 18	116 ± 28	0.15
