# Supplementary material for: Comparing Self-Reported Dietary Intake to Provided Diet during a Randomized Controlled Feeding Intervention: A Pilot Study
Source: Dietetics (Basel). Author manuscript; Available in PMC 2023 Dec 15. (PMC10722558; doi:10.3390/dietetics2040024)
Supplement: Supplemental Table S4 [file NIHMS1950702-supplement-Supplemental_Table_S4.pdf]

Supplemental Table S4. Percent of caloric intake of saturated, monounsaturated, and polyunsaturated fatty acids, stratified by the type of diet. Values are mean ± standard deviation. P-value < 0.05 is bolded.

	**Provided Percent Intake**	**Reported Percent Intake**	**p-value**
**standard diet**			
saturated fat (%)	15.6 ± 4.1	12.7 ± 3.8	0.06
monounsaturated fat (%)	11.0 ± 1.0	10.8 ± 1.8	0.82
polyunsaturated fat (%)	7.4 ± 1.4	8.2 ± 1.5	0.18
**high carbohydrate diet**			
saturated fat (%)	3.8 ± 1.0	6.2 ± 0.8	**7.80E-07**
monounsaturated fat (%)	3.3 ± 0.6	5.7 ± 0.8	**2.14E-09**
polyunsaturated fat (%)	2.4 ± 0.4	3.5 ± 0.8	**0.0004**
**high fat diet**			
saturated fat (%)	27.7 ± 1.3	22.7 ± 3.3	**0.0002**
monounsaturated fat (%)	18.0 ± 1.6	16.8 ± 1.7	0.10
polyunsaturated fat (%)	9.2 ± 1.6	9.6 ± 1.7	0.63
