# Supplementary material for: Comparing Self-Reported Dietary Intake to Provided Diet during a Randomized Controlled Feeding Intervention: A Pilot Study
Source: Dietetics (Basel). Author manuscript; Available in PMC 2023 Dec 15. (PMC10722558; doi:10.3390/dietetics2040024)
Supplement: Supplemental Table S5 [file NIHMS1950702-supplement-Supplemental_Table_S5.pdf]

Supplemental Table S5. Differences between actual and self-reported number of servings of food groups, stratified by experimental diet. P-value ≤ 0.05 highlighted in red. Blanks indicate specific food was not in the diet.

Food group		Standard			HC			HF		
		Provided (servings)	Reported (servings)	p-value	Provided (servings)	Reported (servings)	p-value	Provided (servings)	Reported (servings)	p-value
Fruit	Citrus Juice	1.170	1.029	0.658	1.095	1.144	0.863	0.073	0.037	0.561
	Fruit Juice excluding Citrus Juice	0.076	0.152	0.659	2.203	1.899	0.473	0.037	0.073	0.564
	Citrus Fruit	0.143	0.000	0.327	0.578	0.338	0.073			
	Fruit excluding Citrus Fruit	1.433	1.217	0.548	3.891	2.856	**0.029**	0.165	0.127	0.676
Vegetables	Dark-green Vegetables	1.170	0.589	0.479	1.009	0.568	0.056	1.761	1.115	0.132
	Deep-yellow Vegetables	0.135	0.029	0.139	0.495	0.367	0.331	0.142	0.089	0.411
	Tomato	0.712	1.063	0.214	1.103	1.332	0.424	0.229	0.326	0.534
	White Potatoes	0.046	0.036	0.859	0.291	0.490	0.325	0.162	0.164	0.976
	Other Starchy Vegetables	0.078	0.071	0.951	0.463	0.362	0.521	0.231	0.356	0.411
	Legumes (cooked dried beans)				0.189	0.296	0.223			
	Other Vegetables	0.155	0.257	0.272	1.777	2.504	0.059	0.644	1.130	0.119
	Vegetable-based Savory Snack	1.071	1.277	0.512	0.028	0.028	1.000	1.241	1.302	0.802
Grain	Grains, Flour and Dry Mixes - Whole Grain	0.238	0.286	0.899	0.371	0.444	0.760	0.345	0.707	0.199
	Grains, Flour and Dry Mixes - Refined Grain	0.396	0.876	0.155	0.502	0.481	0.916	0.227	0.328	0.419
	Loaf-type Bread and Plain Rolls - Whole Grain	0.449	0.107	0.300	0.479	0.521	0.873	0.170	0.340	0.378
	Loaf-type Bread and Plain Rolls - Some Whole Grain	0.000	0.214	0.178	2.069	1.095	**0.016**	1.000	0.920	0.783
	Loaf-type Bread and Plain Rolls - Refined Grain	3.107	2.993	0.862	0.638	1.365	**0.028**	1.386	1.429	0.916
	Other Breads (quick breads, corn muffins, tortillas) - Refined Grain	2.714	2.040	0.303	0.904	0.728	0.506	0.517	0.323	0.359
	Crackers - Refined Grain				0.805	0.508	0.058	0.029	0.015	0.561
	Pasta - Refined Grain	1.571	1.976	0.298	0.834	1.298	0.094	0.834	1.636	**0.012**
	Ready-to-eat Cereal (not presweetened) - Whole Grain	0.096	0.088	0.956	0.298	0.376	0.596			

	Cakes, Cookies, Pies, Pastries, Danish, Doughnuts and Cobblers - Some Whole Grain	0.033	0.033	1.000	0.039	0.039	1.000	0.262	0.497	0.101
	Cakes, Cookies, Pies, Pastries, Danish, Doughnuts and Cobblers - Refined Grain	0.326	0.184	0.496	0.413	0.144	**0.012**	0.462	0.266	0.306
	Snack Chips - Refined Grain				0.917	0.627	0.082	0.000	0.034	0.322
**Meats**	Beef				0.316	1.418	**0.000**	1.563	1.624	0.910
	Lean Beef				0.543	0.048	**0.000**			
	Lean Cured Pork	0.146	0.000	0.153	0.028	0.361	**0.049**	0.552	0.518	0.903
	Poultry	0.104	0.000	0.327						
	Lean Poultry	2.454	5.362	**0.001**	1.732	2.934	**0.034**	1.625	2.007	0.495
	Lean Fish - Fresh and Smoked	0.252	0.429	0.725	0.480	0.765	0.422	0.862	1.417	0.334
	Fried Fish - Commercial Entrée and Fast Food							0.000	0.208	0.078
	Cold Cuts and Sausage	0.151	0.101	0.740				1.155	0.946	0.438
	Lean Cold Cuts and Sausage	0.202	0.336	0.628	1.201	1.033	0.715	0.000	0.586	0.075
**Non-meat protein**	Eggs	1.311	1.409	0.788	0.439	0.782	0.053	0.979	1.211	0.385
	Egg Substitute	0.000	0.071	0.327	0.315	0.039	**0.022**			
	Nuts and Seeds	0.292	0.245	0.903						
	Nut and Seed Butters							0.194	0.207	0.949
	Meat Alternatives	1.789	1.905	0.809				0.000	0.122	0.155
**Dairy**	Milk - Whole	0.071	0.000	0.327						
	Milk - Reduced Fat	0.038	0.000	0.178	0.024	0.024	0.985	0.255	0.197	0.462
	Milk - Low Fat and Fat Free	0.000	0.087	0.236	1.894	1.256	**0.003**	0.010	0.057	0.165
	Cheese - Full Fat	1.365	1.187	0.516	0.211	0.467	**0.044**	1.895	1.766	0.424
	Cheese - Reduced Fat	0.466	0.932	**0.045**	0.061	0.150	0.194	0.072	0.239	0.147
	Cheese - Low Fat and Fat Free				0.005	0.000	0.084			
	Yogurt - Sweetened Low Fat	0.050	0.107	0.536	0.031	0.331	**0.005**	0.000	0.097	**0.047**
	Yogurt - Sweetened Fat Free	0.149	0.000	0.178	0.145	0.094	0.525	0.144	0.045	0.169
	Frozen Dairy Dessert	1.055	0.516	0.145	1.455	1.275	0.544	0.123	0.103	0.839
	Frozen Nondairy Dessert	0.041	0.214	0.434	0.286	0.303	0.873			

	Pudding and Other Dairy Dessert				0.081	0.052	0.443	0.030	0.051	0.504
	Cream	0.133	0.068	0.668	0.060	0.058	0.965	0.257	0.162	0.423
**Additives**	Margarine - Regular	0.000	0.143	0.327				0.106	0.063	0.531
	Margarine - Reduced Fat	2.312	3.933	0.062	0.094	1.016	**0.001**	2.671	4.420	0.052
	Oil	0.606	1.313	0.260	0.220	0.233	0.872	0.649	0.464	0.504
	Shortening	0.000	0.065	0.224	0.000	0.195	**0.006**	0.091	0.211	0.436
	Butter and Other Animal Fats - Regular	6.495	2.107	**0.000**	0.310	0.198	0.562	14.226	9.576	**0.001**
	Salad Dressing - Regular	0.262	0.320	0.835	0.000	0.271	**0.000**	0.374	0.330	0.810
	Salad Dressing - Reduced Fat/Reduced Calorie/Fat Free	1.029	0.571	0.141	0.827	0.255	**0.001**	0.138	0.083	0.511
	Sugar	0.466	0.460	0.972	1.670	1.150	0.332	0.063	0.114	0.466
	Sugar Substitute	0.143	0.000	0.327				0.552	1.276	0.196
	Chocolate Candy	0.161	0.163	0.992				0.284	0.314	0.851
	Non-chocolate Candy	0.082	0.035	0.606	0.163	0.271	0.443			
	Sauces and Condiments - Regular	0.166	0.037	0.298	0.035	0.013	0.564	0.110	0.176	0.474
	Sauces and Condiments - Reduced Fat	0.386	0.209	0.563	0.623	0.515	0.599	0.642	0.694	0.815
